# Ecological Role of Bacteria Involved in the Biogeochemical Cycles of Mangroves Based on Functional Genes Detected through GeoChip 5.0

**DOI:** 10.1128/msphere.00936-21

**Published:** 2022-01-12

**Authors:** Shanshan Meng, Tao Peng, Xiaobo Liu, Hui Wang, Tongwang Huang, Ji-Dong Gu, Zhong Hu

**Affiliations:** a Department of Biology, Shantou University, Shantou, Guangdong, People’s Republic of China; b Environmental Science and Engineering Research Group, Guangdong Technion-Israel Institute of Technology, Shantou, Guangdong, People’s Republic of China; c Southern Marine Science and Engineering Guangdong Laboratory (Guangzhou), Guangzhou, Guangdong, People’s Republic of China; University of Wisconsin—Madison

**Keywords:** GeoChip, bacterial community, biogeochemical cycle, functional characterization, mangrove

## Abstract

Mangroves provide a variety of ecosystem services and contribute greatly to the global biogeochemical cycle. Microorganisms play important roles in biogeochemical cycles and maintain the dynamic balance of mangroves. However, the roles of bacteria in the biogeochemical cycles of mangroves and their ecological distribution and functions remain largely uncharacterized. This study thus sought to analyze and compare the ecological distributions and potential roles of bacteria in typical mangroves using 16S rRNA gene amplicon sequencing and GeoChip. Interestingly, the bacterial community compositions were largely similar in the studied mangroves, including Shenzhen, Yunxiao, Zhanjiang, Hainan, Hongkong, Fangchenggang, and Beihai mangroves. Moreover, *gamma-proteobacterium_uncultured* and *Woeseia* were the most abundant microorganisms in the mangroves. Furthermore, most of the bacterial communities were significantly correlated with phosphorus levels (*P < *0.05; −0.93 < *R* < 0.93), suggesting that this nutrient is a vital driver of bacterial community composition. Additionally, GeoChip analysis indicated that the functional genes *amyA*, *narG*, *dsrA*, and *ppx* were highly abundant in the studied mangroves, suggesting that carbon degradation, denitrification, sulfite reduction, and polyphosphate degradation are crucial processes in typical mangroves. Moreover, several genera were found to synergistically participate in biogeochemical cycles in mangroves. For instance, *Neisseria*, *Ruegeria*, *Rhodococcus*, *Desulfotomaculum*, and *Gordonia* were synergistically involved in the carbon, nitrogen, and sulfur cycles, whereas *Neisseria* and Treponema were synergistically involved in the nitrogen cycle and the sulfur cycle. Taken together, our findings provide novel insights into the ecological roles of bacteria in the biogeochemical cycles of mangroves.

**IMPORTANCE** Bacteria have important functions in biogeochemical cycles, but studies on their function in an important ecosystem, mangroves, are still limited. Here, we investigated the ecological role of bacteria involved in biogeochemical cycles in seven representative mangroves of southern China. Furthermore, various functional genes from bacteria involved in biogeochemical cycles were identified by GeoChip 5.0. The functional genes associated with the carbon cycle (particularly carbon degradation) were the most abundant, suggesting that carbon degradation is the most active process in mangroves. Additionally, some high-abundance bacterial populations were found to synergistically mediate key biogeochemical cycles in the mangroves, including *Neisseria*, Pseudomonas, Treponema, *Desulfotomaculum*, and *Nitrosospira*. In a word, our study gives novel insights into the function of bacteria in biogeochemical cycles in mangroves.

## INTRODUCTION

Biogeochemical cycles are critical components of ecosystem dynamics and contribute to the degradation of refractory organic materials as well as the recycling of nutrients, toxic elements, carbon, nitrogen, sulfur, and phosphorus. Biogeochemical cycles can be either directly or indirectly altered by human activities. Direct effects include changes in the biological, chemical, and physical properties and processes of the environment (1). However, global warming and climate change may threaten the balance of biogeochemical cycles (2). For example, global warming might lead to the loss of large organic carbon stocks in soils (3). Mangroves store larger amounts of carbon (up to 1,023 Mg/ha) than other major global forests (4). However, global mangrove carbon storage decreased by 158.4 metric tons from 1996 to 2016 (5). Furthermore, nitrogen (N) and phosphorus (P) delivery along the land-water continuum are increasing due to climate change. Climate change is likely to increase terrestrial biomass delivery into water bodies and accelerate aquatic biomass production and turnover, thereby potentially increasing the magnitude and frequency of nutrient (P and N) release events (6, 7). Additionally, the sulfur cycle promotes iron deposition and further phosphorus release in freshwater ecosystems. The recent rise in the global sea level threatens to disrupt coastal wetlands, thus altering biogeochemical cycling in mangrove ecosystems (8). Moreover, several studies have indicated that bacteria play an important role in biogeochemical cycles. For example, particle-associated bacteria seem to play a much more important role in biogeochemical cycles than free-living bacteria (9).

Bacteria are present in almost all ecosystems, both terrestrial and aquatic, and play crucial ecological roles (10). For example, bacteria mediate the mineralization of labile carbon (C) (11). Moreover, anammox bacteria play a vital role in the nitrogen cycle (12), and rhizobia are involved in nitrogen fixation (13). Bacteria affect local and global biogeochemical cycles by absorbing organic carbon and nutrients (14, 15), and therefore, the study of these microorganisms is key to understanding ecosystem dynamics.

Mangroves are intertidal wetlands that play crucial ecological roles in tropical and subtropical coastlines worldwide (16). These ecosystems have immense ecological importance due to not only their biodiversity but also their increasing association with human activities (17). Furthermore, mangroves also provide several ecosystem services, including the amelioration of wind effects and coast protection (18, 19). Recently, 16S rRNA gene amplicon sequencing studies have substantially contributed to our knowledge of the phylogeny and community structure of mangrove bacteria (10, 20). Additionally, previous studies have demonstrated that mangroves are uniquely rich in microbial diversity, which in turn contributes greatly to their ecosystem dynamics (21, 22). Another study reported that biogeochemical processes were impacted by many factors such as human activities, microbes, and viruses (23). Therefore, disruption of natural biogeochemical cycles would result in insurmountable damage. Recent studies have reported that mangroves are important carbon sinks (24, 25). Mangroves contribute 10% to 15% (24 Tg C year^−1^) and 10% to 11% of carbon to oceans and terrestrial ecosystems, respectively (26). Furthermore, large quantities of organic matter may accumulate in mangroves, which promotes microbial growth and increases biodiversity. Other studies have demonstrated that biogeochemical cycles are largely driven by microbial activity (27). For example, the dominance of communities involved in the nitrogen cycle and CO_2_ fixation has been found to vary between drought and rewetting cycles (28). Moreover, Pseudomonas, *Stenotrophomonas*, and *Serratia* effectively removed nitrogen in volcanic scoria (29). Bacterial communities can also affect biogeochemical cycles (30). For example, *Syntrophobacter*, *Sulfurovum*, *Nitrospira*, and *Anaerolinea* could potentially drive the coupling of the carbon, nitrogen, and sulfur cycles in the Yunxiao (YX) mangrove (10). However, there is a lack of research on other mangroves. Furthermore, 2% of the world’s mangroves are located in China. The southeast coast is the main distribution area of mangroves in China. Therefore, we selected seven research areas along the southern coastline of China that covered the main distribution of mangroves (31, 32).

Here, we sought to investigate the ecological distribution and potential roles of bacteria in the biogeochemical cycles of representative mangroves of southern China based on the expression of functional genes detected by GeoChip analysis to (i) characterize the distribution of functional genes in south China mangroves; (ii) explore the key ecological functions involved in the carbon, nitrogen, sulfur, and phosphorus cycles of typical mangroves of China; and (iii) identify bacteria that synergistically participate in biogeochemical cycles in typical mangroves of China. Therefore, the findings of this study provide novel insights into the ecological roles of bacteria in biogeochemical cycles in mangroves, thus establishing a theoretical basis for the development of strategies to safeguard the ecological stability of mangroves.

## RESULTS

### Correlation between bacterial communities and physicochemical parameters.

**FIG 1:**
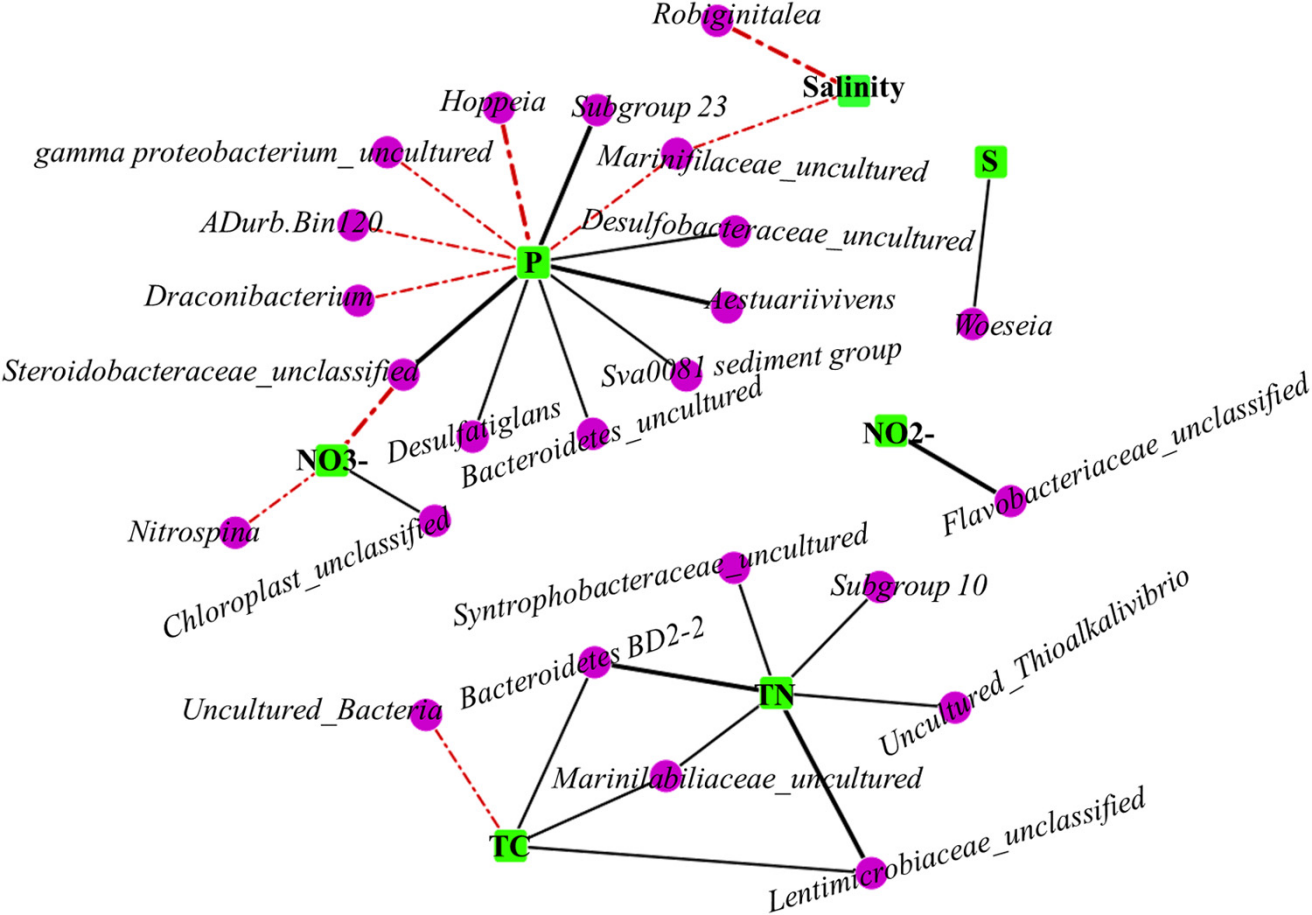
Correlation network between bacterial communities and environmental factors. Pink dots, bacterial communities; green squares, environmental factors; red lines, negative correlation; black lines, positive correlation. Thicker lines indicate *P* values of <0.001.

The bacterial community distribution in the studied mangroves was described in our previous study (33). Concretely, our previous findings demonstrated that the bacterial community structures of the different mangroves were largely similar. Many bacteria in these mangroves were unclassified and uncultured; however, members of *gamma proteobacterium_uncultured* and the *Woeseia* genus were abundant in most of the studied mangroves (see Fig. S1 in the supplemental material). To explore the interactions between bacterial communities and physicochemical parameters, a network was constructed and analyzed. There were more interactions between P and bacterial communities. For example, *Hoppeia*, *gamma proteobacterium_uncultured*, *ADur.Binl 20*, *Marinifilaceae_uncultured*, and *Draconibacterium* were negatively correlated with P (*P* < 0.05; −0.82< *R*< 0.86). *Marinifilaceae_uncultured* also exhibited a negative correlation with salinity. Particularly, *Hoppeia* had a significant negative correlation with P. Moreover, subgroup 23, *Desulfobacteraceae_uncultured*, *Aestuariivivens*, the Sva0081 sediment group, *Bacteroidetes_uncultured*, *Steroidobacteraceae_unclassified*, and *Desulfatiglans* were positively correlated with P. *Steroidobacteraceae_unclassified* and subgroup 23 had a significant positive correlation with P, whereas *Steroidobacteraceae_unclassified* had a negative correlation with NO_3_^−^ (nitrate). Furthermore, *Bacteroidetes* BD2-2, *Marinilabiliaceae_uncultured*, and *Lentimicrobiaceae_unclassified* had a positive correlation with total nitrogen (TN) and total carbon (TC). The correlation between *Lentimicrobiaceae_unclassified* and *Bacteroidetes* BD2-2 with TN was extremely significant. S exhibited a positive correlation only with *Woeseia*, which was most abundant in the Fangchenggang mangrove (FCG-S) (Fig. S1). NO_2_^−^ (nitrite) had a significant positive correlation with *Flavobacteriaceae_unclassified*. Moreover, there was a significant negative correlation between salinity and *Robiginitalea*. Interestingly, *Marinilabiliaceae_uncultured* was not only significantly positively correlated with TN but also significantly negatively correlated with salinity (Fig. 1).

10.1128/msphere.00936-21.1 FIG S1 Composition and abundance of bacterial communities in typical mangroves. Download FIG S1, TIF file, 0.3 MB. Copyright © 2022 Meng et al. 2022 Meng et al. https://creativecommons.org/licenses/by/4.0/ This content is distributed under the terms of the Creative Commons Attribution 4.0 International license.

### Ecological functions of genes detected in south China mangroves.

**FIG 2:**
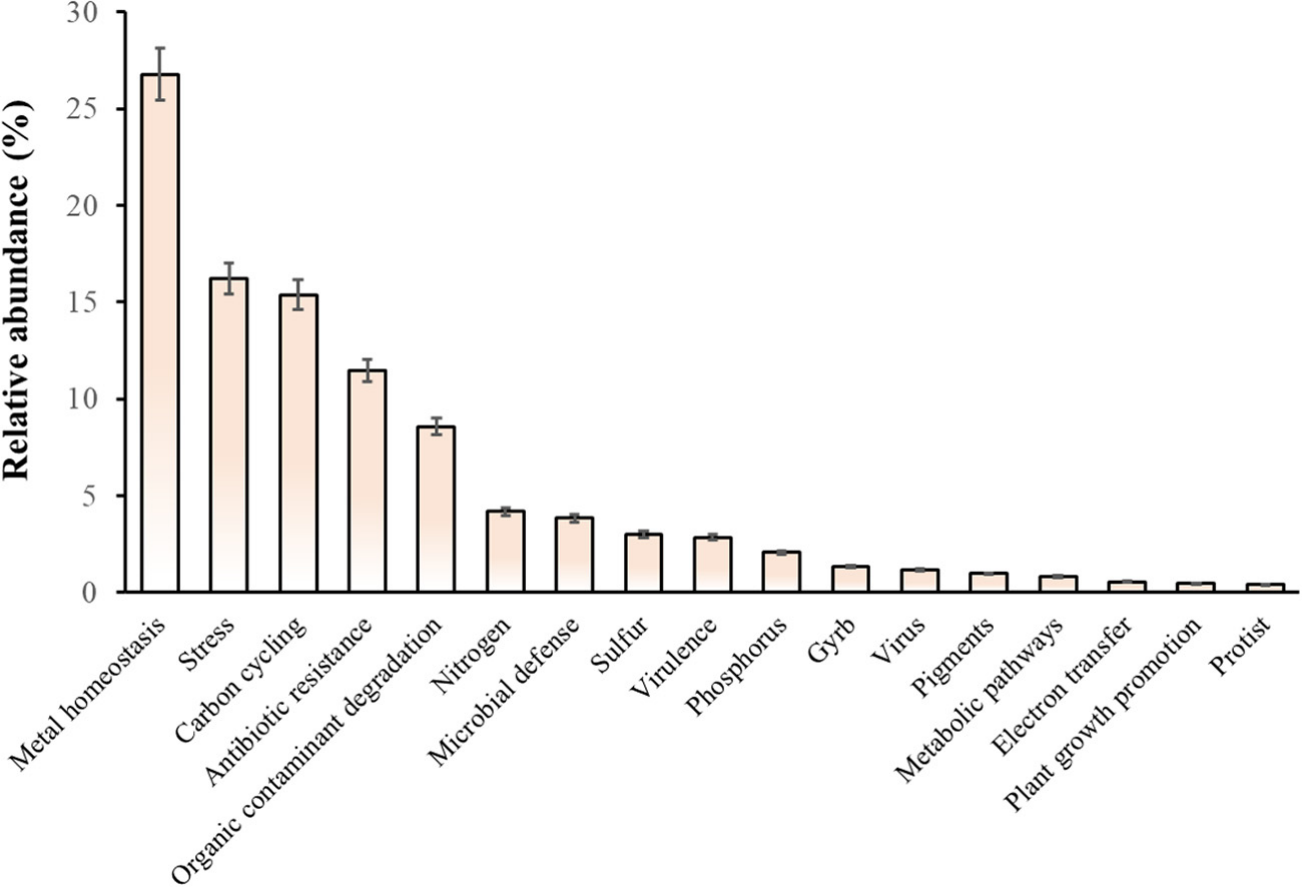
Relative abundances of genes linked to 17 ecological functions in typical mangroves of China.

A total of 17 ecological functions were detected in the studied mangroves, most of which (over 20%) were associated with metal homeostasis. Particularly, these ecological functions were mostly associated with metal homeostasis, stress, the carbon cycle, antibiotic resistance, organic contaminant degradation, and the nitrogen cycle, whereas electron transfer, plant growth promotion, and protists were limited (Fig. 2). Biogeochemical cycles are very important in maintaining the stability of mangroves, particularly the carbon, nitrogen, sulfur, and phosphorus cycles. Furthermore, the abundance of carbon cycle-associated genes exceeded 15%, whereas the abundance of those associated with the nitrogen cycle reached 5%. In contrast, the abundance of sulfur cycle and phosphorus cycle genes was <5% (Fig. 2).

### (i) Carbon cycle genes and ecological functions.

**FIG 3:**
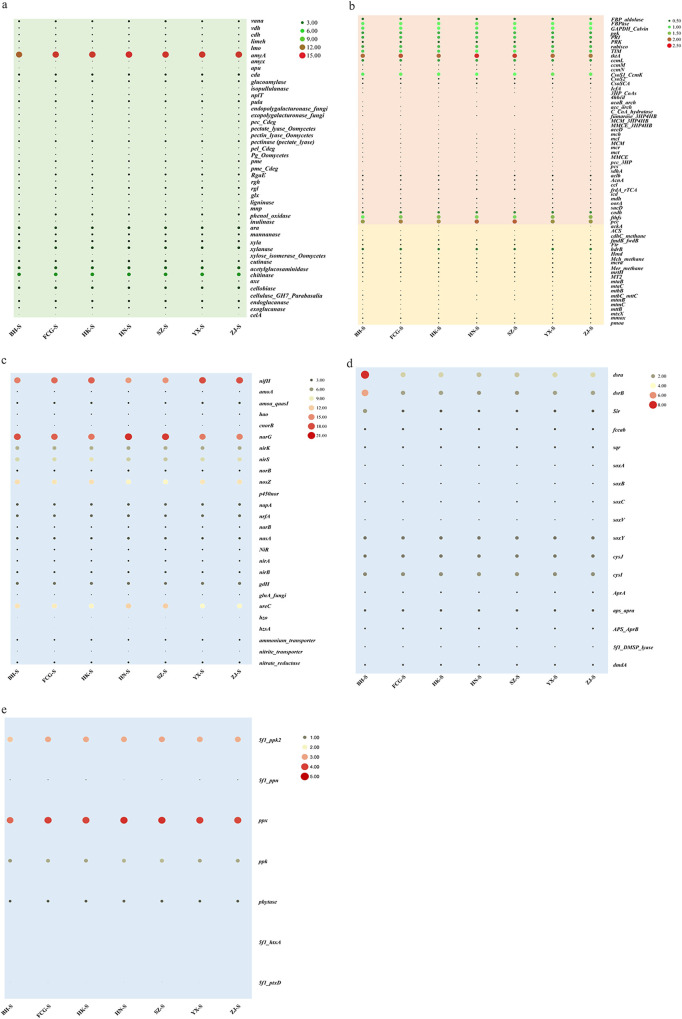
Relative abundances of functional genes in biogeochemical cycling. (a) Carbon degradation; (b) carbon fixation (red) and methane (yellow); (c) nitrogen cycling; (d) sulfur cycling; (e) phosphorus cycling. FBP, fructose 1,6 bisphosphate aldolase; GAPDH, glyceraldehyde-3-phosphate dehydrogenase; PRK, phosphoribulokinase; *cnorB*, genes encoding the nitric oxide reductase catalytic subunit; *5f1_htxA*, genes determining phosphorus oxidation; *5f1_ptxD*, genes determining phosphorus oxidation.

Carbon cycle processes included carbon degradation, carbon fixation, and methane metabolism. Furthermore, the relative abundance of functional genes associated with carbon degradation was generally high (69%) in the mangroves, whereas that of genes associated with methane metabolism was the lowest (3%) (Fig. S2A). A total of 13 processes were linked to carbon degradation (Fig. S2B), among which functional genes associated with starch degradation were the most abundant (26%). These starch-degrading functional genes included the *amyA*, *amyX*, *apu*, *cda*, glucoamylase, isopullulanase, *nplT*, and *pulA* genes. Among these, *amyA* was the most abundant in starch degradation and even carbon degradation, whereas *apu* was the least abundant in starch degradation (Fig. 3a). Moreover, the chitinase gene, which is involved in chitin degradation, was also abundant in the studied mangroves (Fig. 3a). In carbon fixation, the genes with the highest relative abundances were those associated with the Calvin cycle (Fig. S2B), whereas those associated with the 3-hydroxypropionate/4-hydroxybutyrate cycle had the lowest relative abundances. Moreover, *tktA* was most abundant in carbon fixation (Fig. 3b). In the methane cycle, the genes with the highest relative abundances were those associated with methanogenesis (Fig. S2B). *harB* was the most abundant gene associated with the methane cycle (Fig. 3b). Interestingly, the numbers of genes involved in the carbon cycle were similar among various mangroves.

10.1128/msphere.00936-21.2 FIG S2 (A) Relative abundance of genes linked to carbon cycle functions. (B) Relative abundance of genes linked to carbon cycle metabolic processes (carbon degradation, carbon fixation, and methane). Download FIG S2, TIF file, 0.3 MB. Copyright © 2022 Meng et al. 2022 Meng et al. https://creativecommons.org/licenses/by/4.0/ This content is distributed under the terms of the Creative Commons Attribution 4.0 International license.

### (ii) Nitrogen cycle genes and ecological functions.

Functional genes involved in the processes of denitrification, nitrogen fixation, ammonification, assimilatory N reduction, dissimilatory N reduction, nitrification, assimilation, N assimilation, and anammox were detected in this study. Genes associated with denitrification were the most abundant (45%) (Fig. S3). Moreover, nitrogen fixation accounted for 16.33%, whereas anammox accounted for only 0.36% (Fig. S3). Additionally, the functional genes involved in denitrification, including *cnorB*, *narG*, *nirK*, *nirS*, *norB*, *nosZ*, and *p450nor*, were found to be highly diverse. Among these, *narG* was the most abundant denitrification gene (Fig. 3c). However, the abundances of the above-mentioned functional genes were largely similar among the mangroves. The *nifH* gene, which is involved in nitrogen fixation, was also abundant in the studied mangroves. Regarding ammonification, the *ureC* gene was the most abundant, whereas the most abundant gene associated with assimilatory N reduction was *nraB*. The most abundant nitrification gene was *amoa_quasI*. The nitrate reductase gene was the most abundant in the process of N assimilation, whereas the *hzo* gene was the least abundant in the nitrogen cycle (Fig. 3c).

10.1128/msphere.00936-21.3 FIG S3 Relative abundance of functional genes linked to nitrogen cycle processes. Download FIG S3, TIF file, 0.1 MB. Copyright © 2022 Meng et al. 2022 Meng et al. https://creativecommons.org/licenses/by/4.0/ This content is distributed under the terms of the Creative Commons Attribution 4.0 International license.

### (iii) Sulfur cycle genes and ecological functions.

Several genes associated with sulfur cycle-associated processes were detected in the mangroves, including dimethylsulfoniopropionate (DMSP) degradation, sulfite reduction, reduction, sulfur assimilation, other sulfur cycles, adenylylsulfate reductase, and sulfite oxidation (Fig. S4). The genes involved in sulfite reduction were the most abundant (48.5%), whereas those involved in DMSP degradation exhibited the lowest abundance (2.8%) (Fig. S4). Interestingly, functional genes related to sulfite reduction, including *dsrA*, *dsrB*, and *sir*, were highly abundant in these mangroves, indicating strong sulfite reduction activity in the mangroves. Particularly, *dsrB* was the most abundant in the Beihai (BH) mangrove (Fig. 3d). Moreover, the functional genes *soxV* (sulfur oxidation), *cysJ* (reduction), and *cysI* (other sulfur cycles) also exhibited high abundances in the mangroves (Fig. 3d), suggesting high sulfur oxidation and reduction activities in the mangroves.

10.1128/msphere.00936-21.4 FIG S4 Relative abundance of genes associated with sulfur metabolic processes in mangroves. Download FIG S4, TIF file, 0.1 MB. Copyright © 2022 Meng et al. 2022 Meng et al. https://creativecommons.org/licenses/by/4.0/ This content is distributed under the terms of the Creative Commons Attribution 4.0 International license.

### (iv) Phosphorus cycle genes and ecological functions.

Regarding phosphorus cycle genes, our study detected functional genes associated with polyphosphate degradation (76.3%), polyphosphate synthesis (15.3%), phytic acid hydrolysis (7.9%), and phosphorus oxidation (0.46%). Those associated with polyphosphate degradation were the most abundant (Fig. S5), of which the *ppx* gene was the most abundant. In contrast, *5f1_htxA* and *5f1_ptxD* (phosphorus oxidation) were the least abundant (Fig. 3e), suggesting that the mangroves had low phosphorus oxidation activity.

10.1128/msphere.00936-21.5 FIG S5 Relative abundance of genes associated with phosphorus metabolic processes in mangroves. Download FIG S5, TIF file, 0.1 MB. Copyright © 2022 Meng et al. 2022 Meng et al. https://creativecommons.org/licenses/by/4.0/ This content is distributed under the terms of the Creative Commons Attribution 4.0 International license.

### Bacteria synergistically participate in biogeochemical cycles in mangroves.

**FIG 4:**
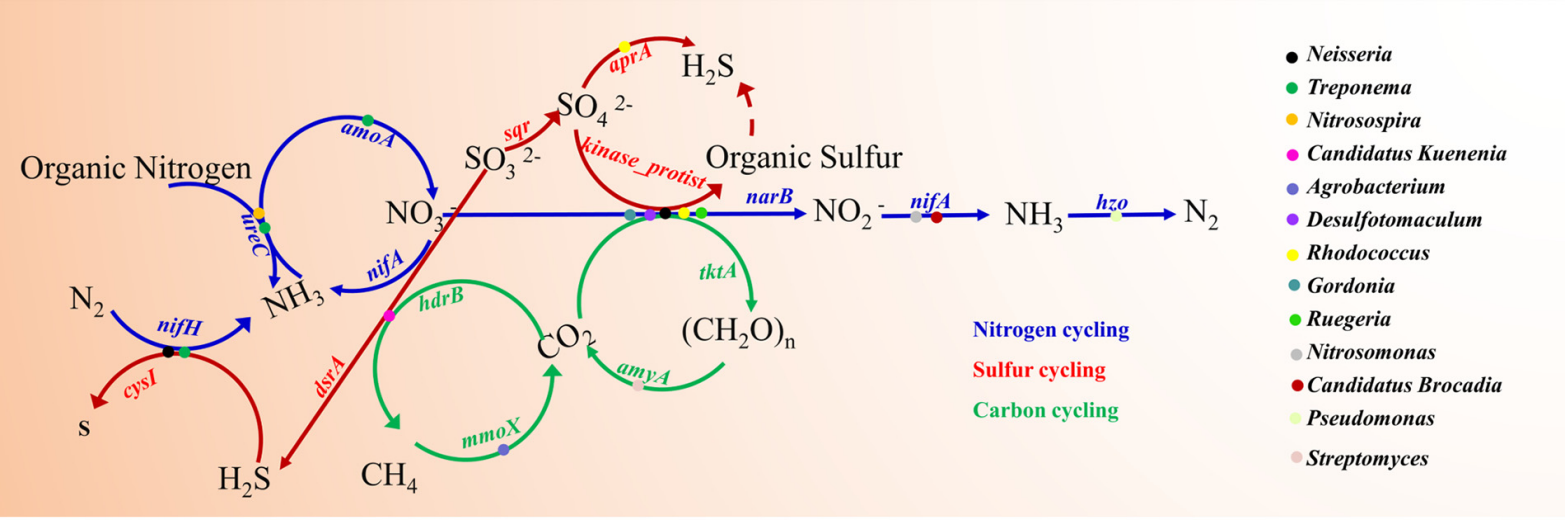
Major functional genes and functional communities involved in biogeochemical cycles in typical mangroves. Dots, bacterial community (only functional genes and communities with high abundances are shown).

More than one bacterial species is commonly involved in any given biogeochemical process. In the studied mangroves, a total of 12 highly abundant bacteria were known to synergistically participate in biogeochemical cycles, including *Neisseria*, Treponema, *Nitrosospira*, “*Candidatus* Kuenenia,” *Agrobacterium*, *Desulfotomaculum*, *Rhodococcus*, *Gordonia*, *Ruegeria*, *Nitrosomonas*, “*Candidatus* Brocadia,” and Pseudomonas (Fig. 4). *Neisseria* and Treponema are both involved in sulfur reduction and anammox, whereas Treponema also participates in ammonification and nitrification. *Neisseria* is also involved in denitrification. Furthermore, *Neisseria* and Treponema were both linked to the sulfur and nitrogen cycles. “*Candidatus* Kuenenia,” *Desulfotomaculum*, *Rhodococcus*, *Gordonia*, and *Ruegeria* potentially drive the carbon, nitrogen, and sulfur cycles. Moreover, some processes were carried out by unique bacteria. For instance, *Agrobacterium* was involved in methane oxidation in the studied mangroves. In contrast, *Nitrosomonas*, “*Candidatus* Brocadia,” and Pseudomonas, which are involved in denitrification, were more highly abundant. Additionally, certain highly abundant functional genes could be linked to specific processes. For example, the highly abundant *hdrB*, *mmoX*, *amyA*, and *tktK* genes are involved in the carbon cycle, whereas *nifH*, *ureC*, *nifA*, and *amoA* participate in the nitrogen cycle (Fig. 4).

## DISCUSSION

In this study, the bacterial community of the studied mangroves was primarily represented by members of *gamma proteobacterium_uncultured* and the *Woeseia* genus. However, unclassified and uncultured bacteria accounted for a sizeable proportion of the bacterial community, suggesting that mangroves harbor a wide variety of novel bacteria. Some studies have demonstrated that *gamma proteobacterium_uncultured* participates in biogeochemical cycles in various environments such as factories and soil (34, 35). Furthermore, recent studies have indicated that *gamma proteobacterium_uncultured* has a negative correlation with P. In fact, most bacteria also have a correlation with P (Fig. 1), suggesting that bacteria are primarily driven by P. The genus *Woeseia* belongs to the *Gammaproteobacteria* subgroup, as described previously by Zhang et al. (36), and was highly abundant in the Fangchenggang mangrove. *Woeseia* possesses a repertoire of genes for hydrocarbon degradation, as described previously by Bacosa et al., suggesting that this genus is linked to the carbon cycle (37). However, this genus was more significantly correlated with S in this study (*P < *0.01) (Fig. 1), suggesting that it could be involved in the sulfur cycle. Additionally, Liu et al. proposed *Steroidobacteraceae* as a new family (38), and many unclassified members of this family were found in this study. These bacteria exhibited a positive correlation with P and a negative correlation with NO_3_^−^ in the studied mangroves (Fig. 1). This indicated that the mangroves were rich in novel microbial resources, which was consistent with the observations of Li et al. (39). Some bacterial communities that have also been correlated with other physiochemical parameters (TN, TC, and NO_2_^−^) are thought to be closely related to key biogeochemical cycles. For example, *Marinifilaceae_uncultured*, which reportedly utilizes a wide range of carbon sources (40), was correlated with TN and TC in the studied mangroves (Fig. 1). *Flavobacteriaceae* have been reported to utilize diverse carbon sources in marine environments, suggesting a correlation with the carbon cycle (41). However, *Flavobacteriaceae_unclassified* was significantly correlated with NO_2_^−^ in our study, suggesting that it may be involved in the nitrogen cycle. Furthermore, salinity is an important driver of global bacterial community structures (42). Mangroves often exhibit extremely high salinities, and these conditions shape the local bacterial communities (43, 44). In our study, *Marinifilaceae_uncultured* and *Robiginitalea* were negatively correlated with salinity (Fig. 1), indicating that salinity mainly affects their distribution.

Many functional genes associated with metal homeostasis were identified in a previous study (33). However, our study determined that mangroves are uniquely rich in functional genes associated with biogeochemical cycles (e.g., the carbon, nitrogen, sulfur, and phosphorus cycles) via GeoChip 5.0 analysis. GeoChip is a functional gene array that enables the analysis of the functional diversity, composition, structure, and metabolic potential/activity of bacteria, which can also be used to establish links between functional genes and microbial communities (45, 46). However, the efficiency of this approach largely depends on the number of probes. GeoChip has been used to detect functional genes in various environments, such as high-arsenic groundwater (47), pastures (48), and freshwater ecosystems (49). Bai et al. studied the functional gene diversity and metabolic potential in the Zhanjiang (ZJ) mangrove using GeoChip 4.0 (50). In our study, the functional genes were detected in the mangroves using GeoChip 5.0, which contains more than three times more probes than GeoChip 4.0 and can thus provide novel insights into the biogeochemical cycles of mangroves. Our results demonstrated that carbon cycle-associated genes were the most active in the studied mangroves. Mangroves sequester large amounts of carbon and can therefore become a significant source of greenhouse gases when disturbed by changes in land use. These ecosystems thus play an important role in climate mitigation (51, 52). Therefore, maintaining the carbon cycle balance in mangroves is of crucial importance. Starch is among the main organic compounds in mangrove sediments and can be degraded into glucose and maltose (53), thus providing a carbon source for microbes to grow. The relative abundance of carbon degradation-associated genes was high in the studied mangroves, particularly those linked to starch degradation (see Fig. S2A in the supplemental material). The high abundance of *amyA*, which participates in the degradation of starch, could help maintain a stable carbon cycle in mangroves while also providing a rich carbon source to support the growth of mangrove microbes. Few studies have characterized the importance of carbon degradation in mangroves, and therefore, our findings provide important insights into mangrove dynamics. Our study demonstrated that carbon degradation was the most active process in the mangroves. Methane is the second most important greenhouse gas after CO_2_. Rising global temperatures and human activities may increase the CH_4_ efflux rate in coastal ecosystems (54). According to Zhang et al., hydrogenotrophic and methylotrophic methanogenesis are the dominant pathways in mangrove sediments, as demonstrated by functional gene analysis (55, 56). In our study, the abundance of functional genes associated with methane metabolism was generally low (Fig. 3d), indicating that the methane cycle processes were less active in the studied mangroves. Among the genes involved in the methane cycle, the relative abundance of *hdrB* was higher than that of *mmoX*, suggesting that methane synthesis was more active than methane oxidation.

Mangroves are also important nitrogen sinks that can remove approximately 6% of anthropogenic nitrogen inputs into the environment (57). Functional genes involved in denitrification, nitrogen fixation, ammonification, assimilatory N reduction, dissimilatory N reduction, nitrification, assimilation, N assimilation, and anammox were detected in the studied mangroves. The denitrification process oxidizes nitrates to nitrogen and it is very important to maintain a dynamic nitrogen balance in mangroves (58). Functional genes associated with denitrification were also highly abundant (Fig. S3), suggesting that denitrification was a highly active process in the mangroves. The *narG* gene, which is involved in denitrification, was highly abundant in the mangroves, indicating that this gene contributed greatly to the nitrogen cycle. Moreover, the *nifH* gene (involved in nitrogen fixation) was also highly abundant in the mangroves (Fig. 3c), indicating that nitrogen fixation was also a vital process in the mangroves. In contrast, *hzo* had the lowest relative abundance. This gene is involved in the anammox process, suggesting that anammox occurs slowly in the mangroves. These results also indicated that the occurrence of denitrification-associated genes might be explained by the availability of nitrite, as reported previously by Luvizotto et al. (59). Moreover, nitrogen fixation contributed substantially to the nitrogen input in mangrove sediments (60). Taken together, our findings indicate that denitrification and nitrogen fixation are dominant in the mangroves, both of which maintain the dynamic balance of nitrogen and further preserve the stability of the biogeochemical cycles of mangroves.

Additionally, functional genes associated with the sulfur cycle were also detected in the mangroves. The ecological functions linked to the sulfur cycle include sulfite reduction, sulfide oxidation, other sulfur cycles, reduction, adenylylsulfate reductase, sulfur assimilation, and DMSP degradation. Compared with other processes of the sulfur cycle, sulfite reduction-associated genes had the highest abundances in the mangroves, whereas those linked to DMSP degradation were the least abundant, suggesting that sulfite reduction was dominant in the studied mangroves, whereas DMSP degradation was slow in the mangroves. Some studies have indicated that DMSP is a major driver of the sulfur cycle and may have a strong impact on climate (61, 62). However, although DMSP degradation is very important in the sulfur cycle, this process was not dominant in the studied mangroves, indicating that the accumulation of DMSP does not drive the sulfur cycle in these ecosystems.

Regarding the phosphorus cycle, the relative abundance of genes associated with polyphosphate degradation was the highest, suggesting that polyphosphate degradation is the main process in the mangroves. The *ppx* gene, which is involved in polyphosphate degradation, was also found to be highly abundant, and therefore, we concluded that the *ppx* gene dominated the polyphosphate degradation process in the mangroves. Interestingly, functions and genes were not significantly correlated within different mangroves.

A wide variety of bacteria have been linked to several biogeochemical cycles (10). In this study, the bacterial community compositions across different mangroves were very similar; however, their relative abundances varied. Furthermore, this study demonstrated that a large number of bacterial species were involved in the biogeochemical cycles of mangroves. Some bacteria have been found to be synergistically involved in biogeochemical processes. For example, *Gordonia*, *Desulfotomaculum*, *Neisseria*, *Rhodococcus*, and *Ruegeria* are mainly involved in carbon fixation, denitrification, and adenylylsulfate reduction in mangroves (Fig. 4). *Ruegeria* is a denitrifying marine bacterium with the potential ability for cyanophycin synthesis (63). We speculate that *Ruegeria* utilizes carbon for growth and oxidizes sulfur into sulfites, after which it oxidizes sulfites to sulfate and hydrogen sulfide in mangroves. Our findings also suggested that *Ruegeria* played crucial roles in the mangrove ecosystem dynamics as it synergistically participated in the carbon and sulfur cycles in the mangrove. *Rhodococcus* is known to reduce hydrogen sulfide but has also been reported to degrade a wide variety of natural and synthetic organic compounds (64). Our results demonstrated that *Rhodococcus* could be involved in the nitrogen cycle in mangroves, suggesting that this genus is likely involved in various functional metabolic pathways in mangrove ecosystems. Furthermore, Treponema was also found to be involved in the nitrogen cycle, as reported in a previous study (65). In our study, Treponema was found to be involved in the nitrogen and sulfur cycles in the studied mangroves. Moreover, both Treponema and *Neisseria* participated in the nitrogen and the sulfur cycles. Treponema and *Nitrosospira* also participated in the nitrogen cycle (Fig. 4), suggesting that Treponema plays an important role in the nitrogen and sulfur cycles in the mangroves. Furthermore, a previous study reported that *Nitrosospira* could be involved in the nitrogen cycle (66). Our findings indicated that *Nitrosospira* was also involved in the sulfur, carbon, and nitrogen cycles in the mangroves, indicating that *Nitrosospira* played a key role in the biogeochemical cycles of the studied mangroves. Additionally, our findings indicated that Pseudomonas participated only in denitrification (nitrogen cycle), as demonstrated by the high abundance of denitrification-associated functional genes. *Agrobacterium* and *Streptomyces* also exhibited high abundances of genes involved in the carbon cycle.

### Conclusion.

This study investigated the community structures and ecological functions of bacteria in seven representative mangroves of southern China. No significant differences in bacterial community composition were observed among the mangroves, suggesting that there were no geographic differences in the functional genes of the studied mangroves. Moreover, the functional genes associated with the carbon cycle (particularly carbon degradation) were the most abundant, suggesting that carbon degradation is the most active process in mangroves. Additionally, some high-abundance bacterial populations were found to synergistically mediate key biogeochemical cycles in the mangroves, including *Neisseria*, Pseudomonas, Treponema, *Desulfotomaculum*, and *Nitrosospira*. These findings thus provide novel insights into the ecological functions of bacteria in mangrove biogeochemical cycles.

## MATERIALS AND METHODS

### Sample collection.

Superficial (0- to 5-cm) sediment samples were collected in February 2018 from seven different southern China mangroves, Beihai (BH), Fangchenggang (FCG), Zhanjiang (ZJ), Hainan (HN), Shenzhen (SZ), Yunxiao (YX), and Hongkong (HK), using sterile plastic tubing. Three samples were collected from each mangrove (the rhizosphere of Kandelia candel). All sediment samples were stored at 4°C and taken back to the laboratory within 48 h after collection. The physiochemical parameters of the mangroves were investigated in our previous study (33). Detailed information is provided in the supplemental material.

### Functional gene analysis.

Functional genes putatively associated with biogeochemical cycles were detected via GeoChip 5.0 analysis as described previously by Meng et al. (33). Total DNA was extracted from mangrove sediment samples as described previously by Zhou et al. (67). Briefly, the samples were freeze-dried in a vacuum freeze-drier. Next, 0.25 g of sediments was weighed from each sample to conduct phenol extractions. The DNA was then evaluated and labeled using random primers specific for functional genes associated with biogeochemical processes. Afterward, the labeled DNA was purified and transferred to a Labconco Centrivap concentrator (Labconco Corp., Kansas City, MO) to dry at 50°C for 45 min. Prior to hybridization, the DNA was incubated at 95°C for 5 min and maintained at 42°C. The labeled DNA was then placed into a hybridization station (Maui; BioMicro Systems, Salt Lake City, UT, USA) and preheated at 42°C for at least 5 min. Finally, optical signals were obtained with a NimbleGen MS200 scanner (Roche, Madison, WI, USA) and then converted to digital signals using ImaGene 6.0 software (Biodiscovery Inc., El Segundo, CA, USA) to obtain the probe signal intensity. Samples were considered positive if spots with a signal-to-noise ratio of >2.0 were detected in at least 2/3 of the replicate sets. The data were then normalized using logarithm transformation, and the mean signal intensity of each sample was determined. Next, each probe’s intensity was normalized to the average intensity of the corresponding sample. Functional gene data (GeoChip 5.0) are provided in the supplemental material.

### 16S rRNA gene sequences.

The primer pair 515F (5′-GTG YCA GCM GCC GCG GTA A-3′) and 806R (5′-GGA CTA CNV GGG TWT CTA AT-3′) was used to amplify hypervariable region 4 (V4) of bacterial 16S rRNA genes. Afterward, clean data were analyzed using QIIME 2 version 2-2020.2 (68). Representative sequences of different operational taxonomic units (OTUs) were aligned with the SILVA SSU database 138 for taxonomy.

### Data analysis.

Correlation was calculated using the Spearman method. Furthermore, an interaction network of bacterial communities and physicochemical parameters in mangrove sediments was constructed using Cytoscape 3.7.2 software (69). Functional gene expression heat maps were created with the TBtools tool kit (70).

### Data availability.

All 16S rRNA gene sequences were submitted to the Sequence Read Archive (SRA) database under accession number PRJNA556990.

10.1128/msphere.00936-21.6 FIG S6 Sampling sites in the south China mangroves. Download FIG S6, TIF file, 0.9 MB. Copyright © 2022 Meng et al. 2022 Meng et al. https://creativecommons.org/licenses/by/4.0/ This content is distributed under the terms of the Creative Commons Attribution 4.0 International license.

10.1128/msphere.00936-21.7 DATA SET S1 Data of functional GeoChip. Download Data Set S1, XLSX file, 3.4 MB. Copyright © 2022 Meng et al. 2022 Meng et al. https://creativecommons.org/licenses/by/4.0/ This content is distributed under the terms of the Creative Commons Attribution 4.0 International license.
