# Supplementary figures and images for: Ecological Role of Bacteria Involved in the Biogeochemical Cycles of Mangroves Based on Functional Genes Detected through GeoChip 5.0
Source: mSphere. 2022 Jan 12;7(1):e00936-21. doi: 10.1128/msphere.00936-21 (PMC8754168; doi:10.1128/msphere.00936-21)

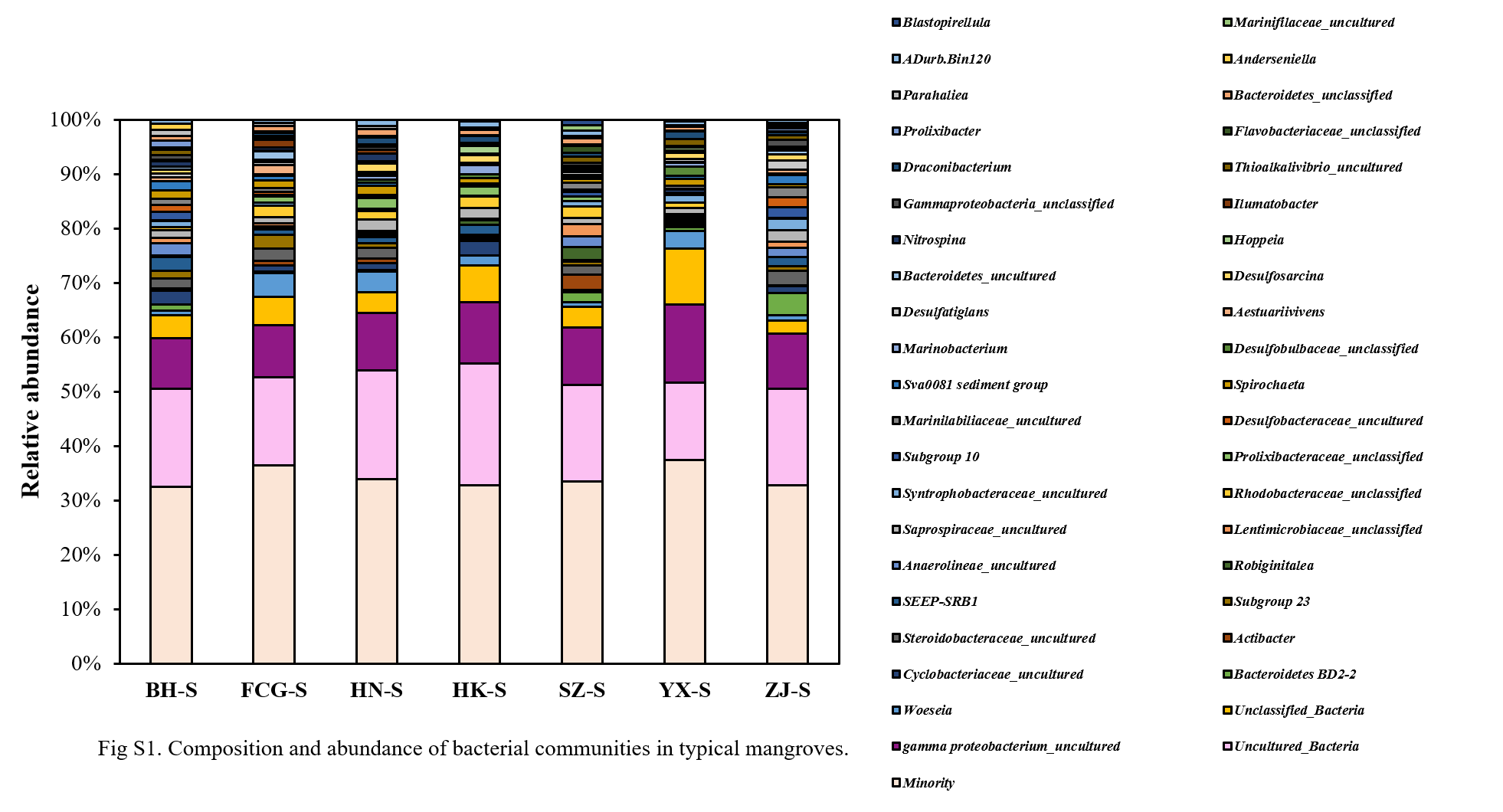

Supplement: FIG S1 [file msphere.00936-21-sf001.tif]

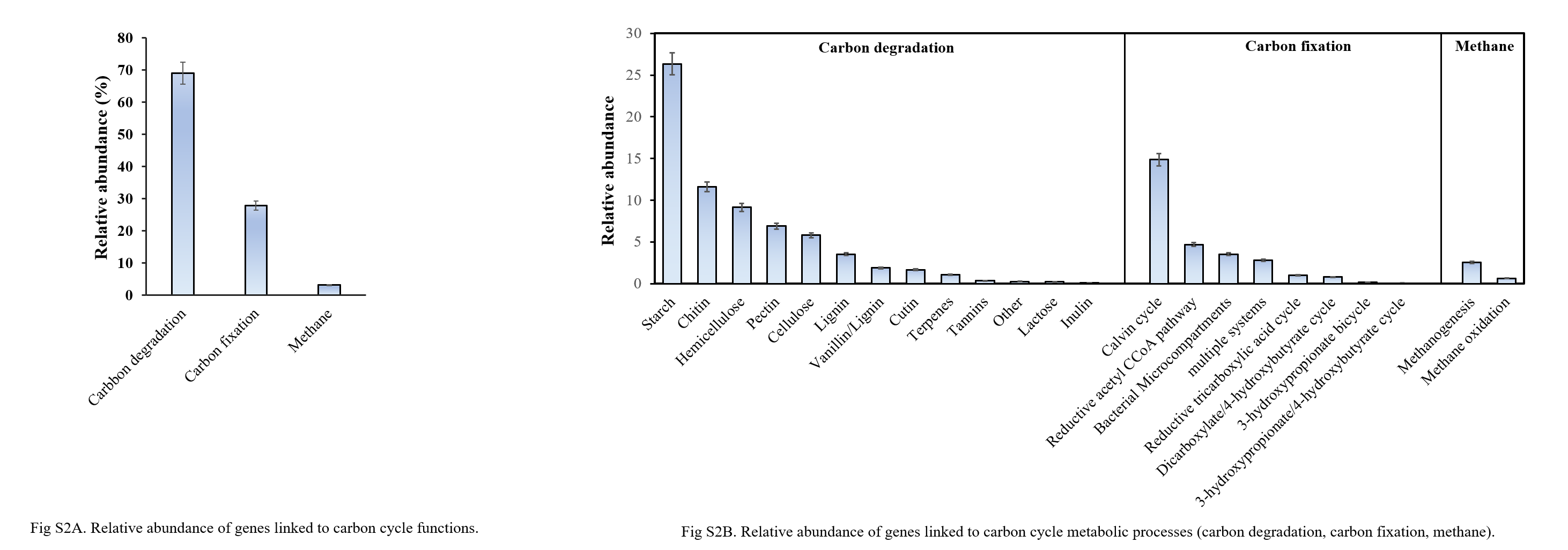

Supplement: FIG S2 [file msphere.00936-21-sf002.tif]

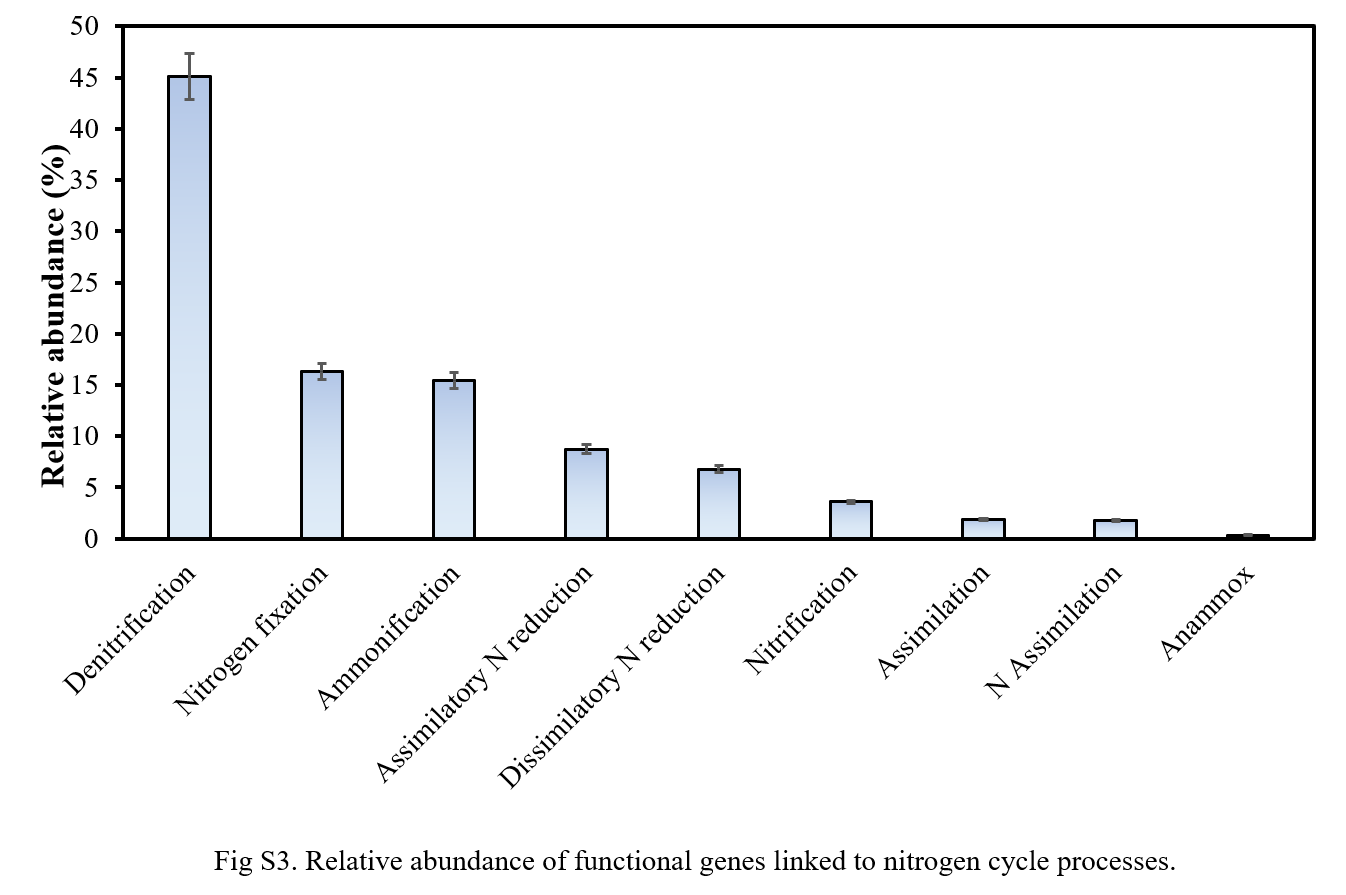

Supplement: FIG S3 [file msphere.00936-21-sf003.tif]

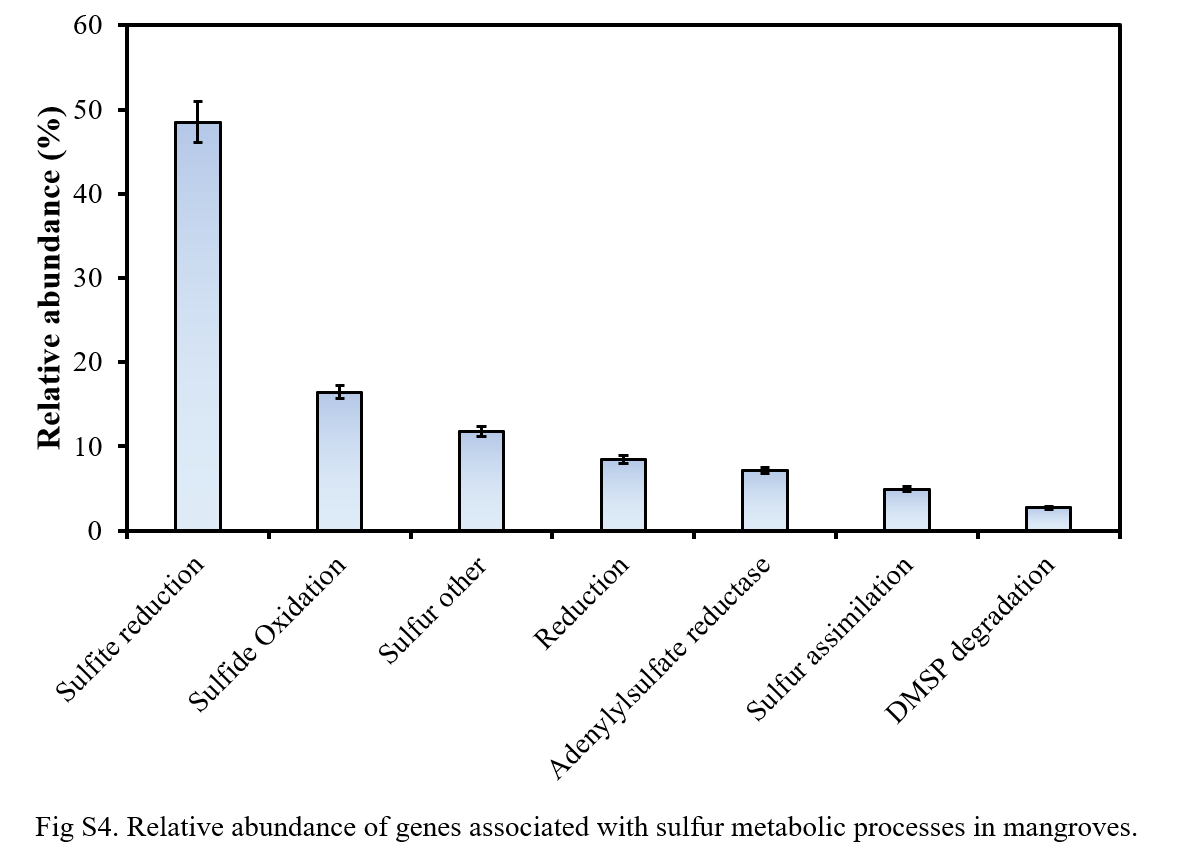

Supplement: FIG S4 [file msphere.00936-21-sf004.tif]

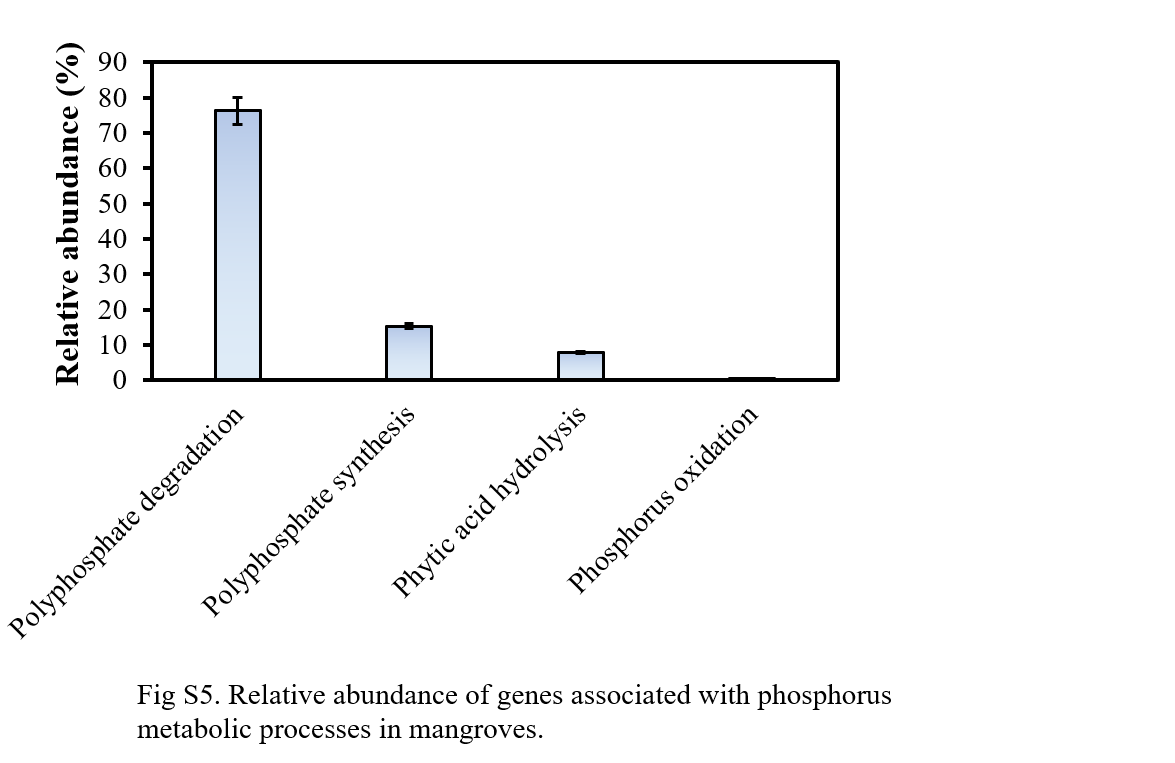

Supplement: FIG S5 [file msphere.00936-21-sf005.tif]

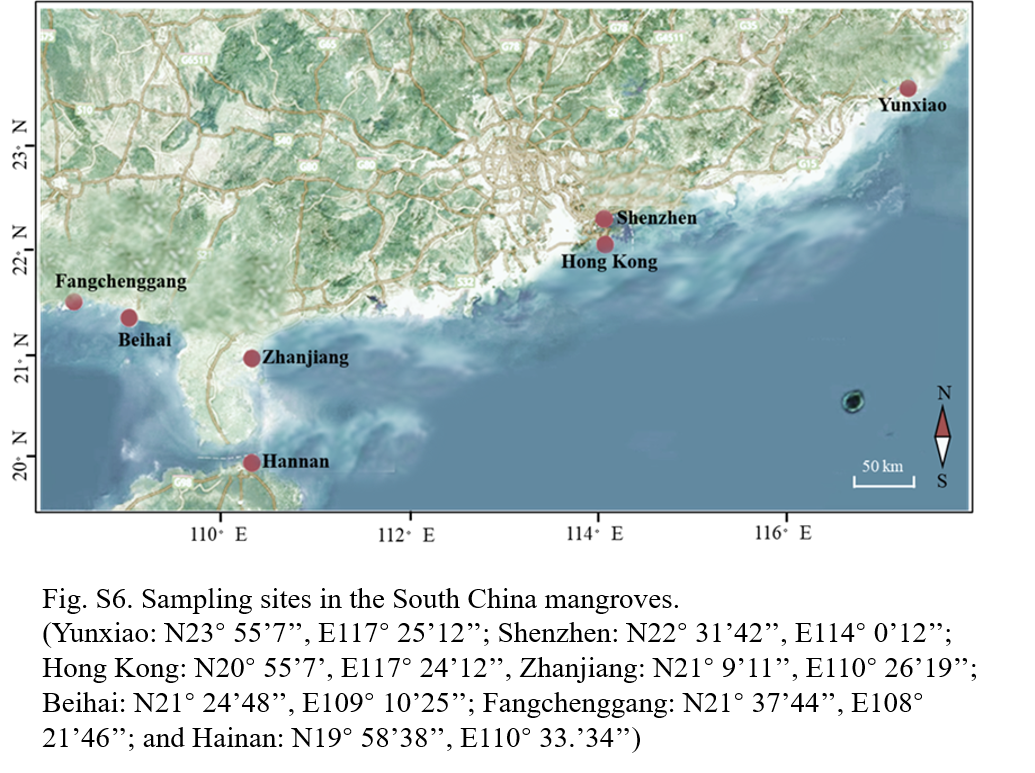

Supplement: FIG S6 [file msphere.00936-21-sf006.tif]
